# A Simple and Valid Method to Calculate Wheelchair Frame Rotation Using One Wheel-Mounted IMU

**DOI:** 10.3390/s23177423

**Published:** 2023-08-25

**Authors:** Marc Klimstra, Melissa Lacroix, Matt Jensen, Joel Greenshields, Daniel Geneau, Patrick Cormier, Ryan Brodie, Drew Commandeur, Ming-Chang Tsai

**Affiliations:** 1 School of Exercise Science, Physical and Health Education, University of Victoria, Victoria, BC V8W 3P2, Canada; 2 Canadian Sport Institute Pacific, Victoria, BC V9E 2C5, Canada; 3 Canadian Sport Institute Ontario, Toronto, ON M1C 0C7, Canada; 4 Wheelchair Rugby Canada, Ottawa, ON K1G 4K3, Canada

**Keywords:** wheelchair, IMU, sport

## Abstract

Wheelchair sports have been using Inertial Measurement Units (IMU) to measure mobility metrics during training, testing and competition. Presently, the most suitable solution to calculate wheelchair speed and frame rotation is the 3IMU method as there is uncertainty about the ability of a one wheel-mounted IMU (1IMU) approach to calculate wheelchair frame rotational kinematics. A new method for calculating wheelchair frame rotational kinematics using a single wheel-mounted IMU is presented and compared to a criterion measurement using a wheelchair-frame-mounted IMU. Goodness-of-fit statistics demonstrate very strong linear relationships between wheelchair frame angular velocity calculated from the wheel-mounted IMUs and a wheelchair-frame-mounted IMU. Root mean square error (RMSE), mean absolute error (MAE) and Bland–Altman analysis show very small differences between the wheelchair frame angular velocity calculated from the wheel-mounted IMUs and the wheelchair-frame-mounted IMU. This study has demonstrated a simple and accurate approach to estimating wheelchair frame rotation using one wheel-mounted IMU during an elite wheelchair athlete agility task. Future research is needed to reexamine and compare wheelchair mobility metrics determined using the 3IMU and 1IMU solutions using this new approach.

## 1. Introduction

Wheelchair sports have been using Inertial Measurement Units (IMU) to measure mobility metrics during training, testing and competition [1,2,3,4,5,6,7,8,9]. This sensor has been valuable to provide metrics specific to many different wheelchair sports, such as rugby, basketball and tennis, and support training interventions and performance tracking [1,5,6]. For example, through IMU sensor data analysis and data reduction techniques, van der Slikke et al. (2016) were able to isolate key kinematic variables during match play in wheelchair basketball across competition levels. Beyond demonstrating the importance of this approach to evaluate wheelchair athlete performance, they demonstrated the value of this measurement tool to provide mobility metrics with the potential to support classification [2,6,10]. As the use of IMUs in wheelchair sports is still developing, there are considerations regarding the best sensor placement, number of sensors and analysis methods to determine accurate kinematics to support accurate wheelchair performance data [1,11,12]. 

**Figure 1:**
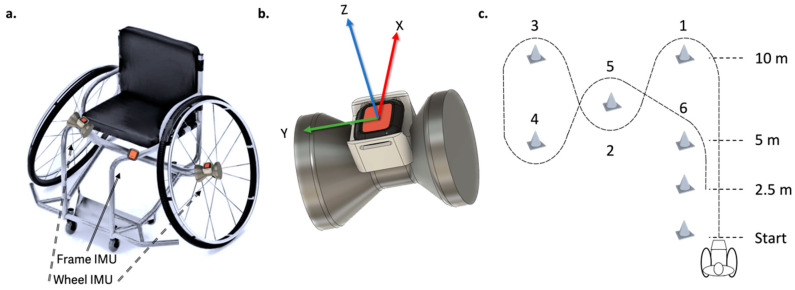
(**a**) Three mounting locations of the IMUs, two on each wheel hub and one on the frame. (**b**) The custom-made mount placed on the wheel hub showing the IMU axis orientation. (**c**) The schematic of the agility task in the left turn variation. The numbers on the task refer to common locations of cones in the agility task.

While there have been many approaches to using IMUs to measure wheelchair mobility performance, presently, the most suitable solution to calculate wheelchair speed and frame rotation is the 3IMU method which requires one IMU mounted on the chair to collect wheelchair rotation and an IMU on each wheel to collect left and right wheel speed [3,4,6,8,11,12]. Wheelchair frame rotational kinematics are critical variables in wheelchair sport performance. Indeed, van der Slikke et al. 2016 isolated that average rotational speed in a curve had an important interaction with athlete classification and competition level in wheelchair basketball (5). Further, for wheelchair court sports purposes, correct wheelchair speed calculations require accurate wheelchair frame rotational estimates [2]. While the 3IMU solution is effective, it can be overly costly and time-consuming to set up three IMUs on each athlete’s chair. As such, there is ongoing research investigating the feasibility of using one wheel-mounted IMU (1IMU) as a suitable, cost-effective and efficient solution to obtain all of the relevant measures of wheelchair performance [11,12]. Wheel speed can be effectively obtained from a single wheel-mounted IMU [8,9]; however, there is uncertainty about the efficacy of a 1IMU approach in obtaining accurate wheelchair frame rotation kinematics [3,11,12]. Presently, the 3IMU approach uses the direct measurement of the wheelchair-frame-mounted IMU gyroscope signal to measure chair rotation. As there is constant change in the orientation of the wheel IMU sensor across multiple axes during normal wheelchair sports movement, determining accurate frame rotation is considered a potentially complex solution, and the 1IMU approach has not been fully indicated to obtain all wheelchair mobility metrics [11,12]. An approach to determine frame rotation from 1IMU is through the use of a Madgwick filter which develops a corrected IMU orientation estimate using quaternion-based sensor fusion [13,14]. However, while this has shown great promise in some investigations [4,11], the current one wheel-mounted IMU approach can result in orientation estimation errors that may not be appropriate for valid wheelchair frame rotation estimates [12]. This may be due to difficulties of the orientation algorithm to properly correct the sensor measurements. Therefore, this approach requires greater exploration before widespread application. Another approach to obtaining wheelchair rotation using 1IMU could be applying a simple trigonometric approach to the solution based on known task, mounting and measurement parameters (Figure 1). For example, as a wheel-mounted IMU is mounted on a wheel hub, one gyroscope axis is constrained to rotate on a wheel plane, while the two other gyroscope axes are constrained to rotate on a wheelchair rotation plane. This may reduce the complexity of the solution to focus primarily on two axes to solve for wheelchair frame rotation. Additionally, as the two accelerometer axes in the wheel rotation plane can be used to calculate the orientation of the gyro axes due to gravity, there is potential to determine and sum the appropriate wheelchair frame rotation components, and the result is a simple yet suitable estimate [3]. 

Therefore, the purpose of this study is to develop and assess a simple mathematical approach to calculating wheelchair frame rotation using a single wheel-mounted IMU. This approach will be compared to a criterion measurement from a wheelchair-frame-mounted IMU for goodness-of-fit statistics to determine its suitability as an effective method to calculate wheelchair frame rotational kinematics in wheelchair sports. 

## 2. Materials and Methods

### 2.1. Participants

Subjects (N = 9) from a national wheelchair rugby program were included in this analysis. 

### 2.2. Testing Protocol

Subjects performed an agility test which involved athletes sprinting through a circuit as quickly as possible (Figure 1c). Trials consisted of 2 circuit variations. In the first variation, subjects performed a 10 m straight sprint at which point the athlete turned left and then turned around a series of cones until returning to the starting position. The athletes then performed a forward and backward start and stop task within the original 5 m to complete the trial. The second variation was a mirror of the first variation with athletes turning to the right after the 10 m sprint and then turning around a series of cones. 

### 2.3. IMU Configuration 

Three Xsens^TM^ dot IMUs (60 Hz) were mounted on the athlete’s chair (Figure 1a). This was similar to the 3IMU setup from previous studies [3]. One IMU was mounted on the chair such that the positive z-axis was pointed vertically upwards, the positive x-axis was pointed forward and the positive y-axis was pointed rightward. One IMU was also mounted on both the left and right wheels such that the positive z- and x-axes projected radially within the plane of wheel rotation and the y-axis was orthogonal to the wheel rotation (Figure 1b). This wheel fixation was accomplished using a custom 3D-printed mounting fixed to the wheel hub (Figure 1b). 

### 2.4. Data Analysis 

All data were analyzed using custom-written Labview^TM^ software (National Instruments^TM^). IMU synchronization was performed using the Xsens^TM^ proprietary synchronization procedure using the Xsens^TM^ dot app. Data were segmented to include ~3 s of data before and after the agility trial. 

#### 2.4.1. Wheelchair Frame Rotation Criterion and Wheel IMU Estimates

Wheelchair-frame-mounted IMU gyroscope z-axis data were corrected for slight vertical orientation discrepancies (due to mounting) with a vector multiplication of the gyroscope data by the Xsens^TM^ native quaternion, represented as a scalar, $q_{w}$, and vector $\vec{q}$ such that $q=\left(q_{w},\vec{q}\right)$

$$v^{\prime }=qvq^{*}$$

where

***v*** = (0,$\vec{v}$) is the velocity quaternion containing the embedded vector $\vec{v}$

q* = ($q_{w}$, $\vec{q}$) is a conjugate quaternion

***v’*** = (0, ${\vec{v}}^{\prime }$) is the rotated vector ${\vec{v}}^{\prime }$

This corrected frame gyroscope z-axis, ***v***’, was used as the criterion for wheelchair frame angular velocity and is equivalent to the calculation of wheelchair frame rotation using the 3IMU method [3]. Gyroscope (rad/s) and accelerometer (g) data from each wheel IMUs were used to calculate wheelchair frame rotation from the left and right mountings separately and compared to the criterion. The following steps describe the process of developing the frame rotation estimate from a single wheel-mounted IMU. This process was replicated for both the left and right wheel mountings separately to obtain 2 unique estimates. 

#### 2.4.2. Correction for Camber Angle 

The camber angle, *θ*, was determined using the offset (to gravity, *g*) of the wheel IMU y-axis accelerometers, $a_{y}$, taken from 2 s of selected data during the first 10 m when the wheel rotation angular velocity was greater than 5 rad/s and there was minimal angular velocity (>0.2 rad/s) in the z and x gyroscope axes. This was done for each of the left and right IMU datasets, respectively.

$$\theta =arctan\left(\frac{a_{y}}{g}\right)$$

All gyroscope and accelerometer data from the wheel IMU were corrected for wheel camber angle by dividing the measurements by the cosine of the camber angle.

$$a=\frac{\underline{a}}{cos\left(\theta \right)}$$

$$\omega =\frac{\underline{\omega }}{cos\left(\theta \right)}$$

where

*a* and *ω* are the corrected acceleration and angular velocity.

$\vec{a}$ and $\vec{\omega }$ are the acceleration and angular velocity vectors.

θ is the camber angle.

#### 2.4.3. Correction for Mounting Misalignment 

**Figure 2:**
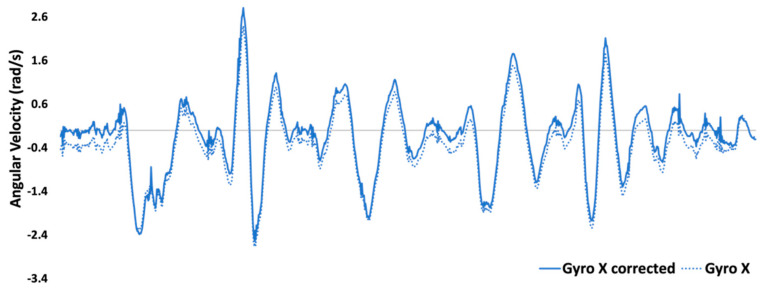
An example of gyroscope x data before and after the mounting misalignment correction described in Section 2.4.3.

A misalignment in IMU mounting on the wheel hub can result in a component of the wheel rotation angular velocity being measured by both the z and x gyroscope data (Figure 2). This wheel rotation component must be removed from both gyroscope z and x before calculating frame rotation components. Two seconds of gyroscope data from all 3 axes were selected during the first 10 m sprint when the wheel rotation angular velocity was greater than 5 rad/s and there was minimal angular velocity (>0.2 rad/s) in the z and x gyroscope axes. Each of the gyroscope z- and x-axes data were divided by the gyroscope y data and averaged for the 2 s to determine the scaling factors for gyroscope *z* (*S_z_*) and gyroscope *x* (*S_x_*).

$$S_{x}=\frac{\sum_{i=1}^{n}\frac{\omega_{x,i}}{\omega_{yxi}}}{n}$$

$$S_{z}=\frac{\sum_{i=1}^{n}\frac{\omega_{z,i}}{\omega_{yxi}}}{n}$$

where *n* is the number of samples defined by 2 s multiplied by the sampling rate/frequency. Corrected gyroscope z (Ωz) and gyroscope x (Ωx) data can be determined separately to remove any component of wheel rotation present in the signal using the scaling factors.

$$\Omega_{x,i}=\omega_{x,i}-S_{x}\omega_{y,i}$$

$$\Omega_{z,i}=\omega_{z,i}-S_{z}\omega_{y,i}$$

where *i* is the datum at each timestamp. Removing this wheel rotation component is a critical step, and without this, an oscillating error can result in the final calculated wheelchair angular velocity, as observed in another study [12]. 

#### 2.4.4. Isolating Gravitational Acceleration in Wheel Accelerometer Data 

The gravitational signal in the wheel-mounted IMU accelerometer z- and x-axes data can be used to determine the orientation of the gyroscope axis. However, due to the mounting and direction of the accelerometer z-axis relative to the wheel hub, this signal contains both the component of gravity relative to the orientation of the IMU as well as the radial acceleration due to the wheel rotation speed. In order to isolate the gravitational component in this axis, it was necessary to remove the radial acceleration. The radial acceleration (*a_r_*) was determined by multiplying the square of *ω_y_* (wheel rotation) signal by the hub radius (*r_w_*). This radial acceleration was subtracted from the accelerometer z signal to isolate the gravitational acceleration in this axis, gravity-free acceleration z (*a*_*z*,*gf*_).

$$a_{r}=\omega_{y}^{2}{\cdot r}_{w}$$

$$a_{z,gf}=a_{z}-a_{r}$$

#### 2.4.5. Determining Orientation of IMU Axis 

At this stage, all gyroscope and accelerometer signals were subject to a 6 Hz low-pass Butterworth filter [5,15]. As the gyroscope z- and x-axes are rotating in the wheel rotation plane, the y-axis (Figure 1b), the component of the wheelchair frame rotation measured by each axis is dependent on the orientation of the axis. Therefore, it was important to determine the exact cartesian orientation of each axis. Fortunately, the accelerometer z- and x-axes measurement of gravity during wheel rotation corresponds to a vertical (positive or negative gravity) and a horizontal (zero gravity) component. Additionally, as the accelerometer z- and x-axes are orthogonal they provide complimentary orientation information; IMU orientation, φ, during wheel rotation could be determined by calculating the angle between the 2 axes [3].

$$\psi_{i}=arctan\left(\frac{a_{z,i}}{a_{x,i}}\right)$$

**Figure 3:**
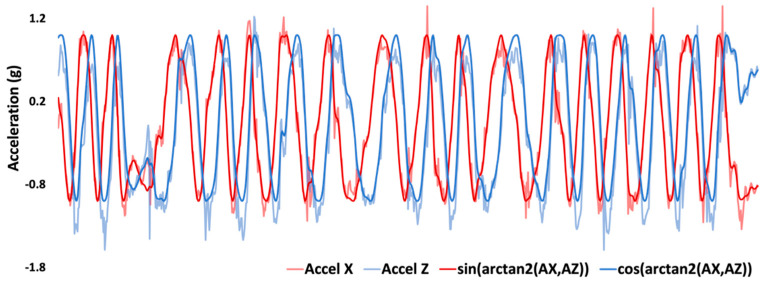
The raw Accelerometer X (red) and Z (blue) shown with the superimposed wheel orientation estimates developed by using the two-argument arctangent function.

A major benefit of the two-argument determination of wheel orientation is that any error signal common to both axes will be minimized. IMU orientation, φ, was then multiplied by sine and cosine to give the orientation of the gyroscope x and gyroscope z, respectively (Figure 3).

#### 2.4.6. Isolating Component of Wheelchair Frame Rotation in Wheel IMU Gyroscope Data

**Figure 4:**
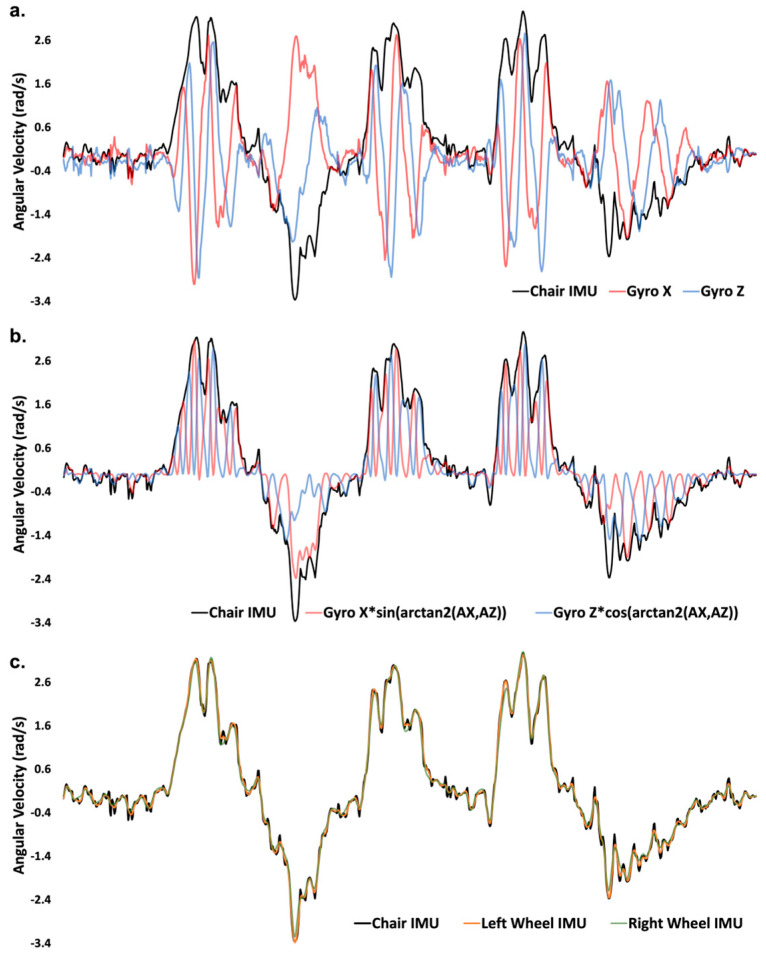
(**a**) Corrected chair-mounted gyroscope Z (frame rotation; black) data and raw gyroscope X (red) and Z (blue) data from one wheel-mounted IMU from one participant during an agility test. (**b**) Same data from 4a with wheel-mounted gyroscope x and z frame rotation components isolated. (**c**) Corrected chair-mounted gyroscope Z (frame rotation; black) with Left (yellow) and Right (green) wheel-mounted IMU estimates of wheelchair frame rotation.

In order to determine the component of wheelchair angular velocity measured by each gyroscope axis, each axis signal was multiplied by the orientation of the axis determined from the respective accelerometer axes in the previous step. The result determined the component of the resultant transverse (wheelchair frame) rotation occurring in each gyroscope axis. Summing the result from each axis returned the resultant wheelchair frame rotation derived from the wheel-mounted IMU (Figure 4).

#### 2.4.7. Average Wheelchair Frame Angular Velocity during Left and Right Turns

The average wheelchair frame angular velocities during left and right turns during the agility tests for all participants were calculated by taking the mean angular velocity of data greater than 0.5 rad/s.

### 2.5. Goodness-of-Fit Statistics

Estimates of wheelchair frame rotation angular velocity derived from left and right wheel-mounted IMUs were compared to measured wheelchair-mounted IMU frame rotation using the coefficient of determination (r^2^), root mean square error (RMSE) and mean absolute error (MAE) from the entire continuous time-series data. Additionally, Bland–Altman analysis was used to demonstrate the agreement between estimates of average wheelchair frame angular velocity during left and right turns from left and right wheel-mounted IMUs and corrected chair-mounted IMU gyroscope z data.

## 3. Results

### 3.1. Goodness-of-Fit Statistics

A summary of the goodness-of-fit statistics is shared in Table 1. The coefficient of determination (r^2^) demonstrated very strong linear relationships between both the chair-mounted IMU gyroscope z-axis and the left and right wheel-mounted IMU frame angular velocity estimates. The RMSE and MAE analyses show very small differences between the chair-mounted frame angular velocity estimates and the left and right frame angular velocity estimates.

### 3.2. Bland–Altman Plot

**Figure 5:**
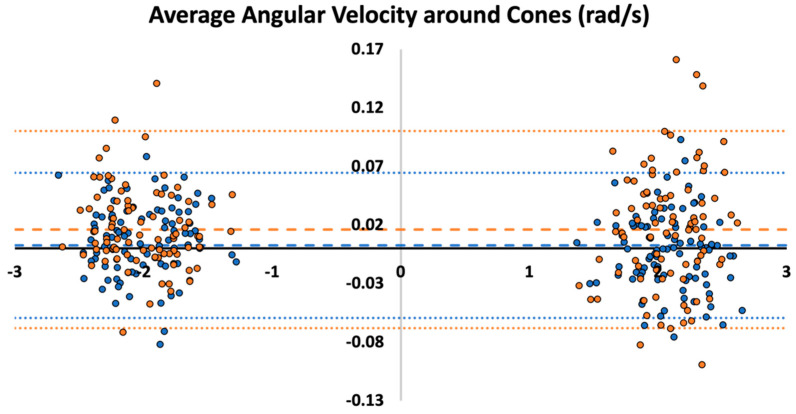
A Bland–Altman plot showing a comparison of the average angular velocities achieved during left (**left-side**) and right (**right-side**) turns against the wheelchair-frame-mounted criterion measure for the left wheel-mounted IMU (orange) and the right wheel-mounted IMU (blue) during an agility test. The dashed lines represent the mean difference between methods, and the dotted lines represent the limits of agreement.

The Bland–Altman plot demonstrates minimal bias and small limits of agreement for average angular velocities during left and right turns in an agility test for both left and right wheel-mounted IMU angular velocity estimates (Figure 5). There was a mean difference of 0.002 rad/s with limits of agreement of 0.064 rad/s and −0.059 rad/s for the right wheel IMU and a mean difference of 0.016 rad/s and limits of agreement of 0.10 rad/s and −0.067 rad/s for the left wheel IMU.

## 4. Discussion

This study has demonstrated a simple and accurate approach to estimating wheelchair frame rotation using one wheel-mounted IMU during an elite wheelchair athlete agility task. Goodness-of-fit statistics and Bland–Altman analysis show strong agreement and minimal error between this method and the criterion wheelchair-frame-mounted IMU measurement. Future research is needed to re-examine and compare wheelchair mobility metrics determined using the 3IMU and 1IMU solutions using this approach.

The use of IMUs in wheelchair sports performance has been gaining credibility as the refinement of this technology is supporting the effective collection of wheelchair sports data [6]. Foundational technology validation research by Pansiot et al. (2011) [16], Mason et al. (2014), van der Slikke et al. (2015) and Shepherd et al. (2016) have set the standard for the use of this technology while Rupf et al. (2021) and Van Dijk et al. (2022) have provided important considerations around technology use and analysis, especially with respect to minimal viable sensors for accurate data collection. As development is continuing, the value of IMUs in collecting wheelchair linear and rotational kinematic data in training and competition has been established [3,5,9] and the potential to use this data to inform important sport decision making, especially around concepts such as classification, is under careful consideration [5,6,10]. Taken together, this puts an important focus on the ability to obtain accurate linear and rotational kinematic measures from an accessible one wheel-mounted sensor solution. The approach presented in this study builds on the work of previous researchers and maintains the standard of kinematic measurement through a simplified approach to analysis that could improve the accessibility of IMU-based wheelchair sport performance data collection.

The strong linear relationship with the criterion in this study is consistent with that demonstrated by Rupf et al. (2021) during a similar agility test using a Madgwick-based 1IMU approach compared to 3IMU estimates. In terms of estimated error, van der Slikke et al. (2015) and van Dijk et al. (2021) reported similar average angular velocity errors of 4.8 deg/s and 4.6 deg/s for 3IMU and 2IMU solutions against an optical motion capture reference. Consistent with Rupf et al. (2021), van Dijk et al. (2021) found that a 1IMU solution had almost double the error (11.2 deg/s) compared to the 2IMU and 3IMU approaches. In the present study, the errors were consistent between the continuous angular velocity comparisons as well as the limits of agreement from the Bland–Altman analysis of the average angular velocity. Impressively, the RMSE from the present frame rotation algorithm was 3.8 deg/s (0.067 rad/s) for the left wheel IMU and 4.2 deg/s (0.074 rad/s) for the right wheel IMU. These results could potentially position this novel approach as equivalent to the 2IMU and 3IMU approaches for frame rotational kinematic estimates. It is important to consider that the present comparison was performed against direct measurements from a frame-mounted IMU as van der Slikke et al. (2015) and van Dijk et al. (2021) used an optical motion capture reference. Future comparisons could apply this present approach as well as other 1IMU approaches against the 3IMU and 2IMU solutions using an optical motion capture reference [3].

Clearly, a 1IMU approach has been shown to have different levels of success in the determination of wheelchair frame angular kinematics when considering this present result and previous studies [4,5]. Using the same Madgwick-based 1IMU approach Rupf et al. (2021) demonstrated high linearity and low error when comparing 1IMU estimates, while van Dijk et al. (2021) found larger angular velocity errors for the 1IMU solution than a 2IMU (wheelchair frame, wheel) solution. Van Dijk et al. (2021) also observed a large oscillating error occasionally occurring in the 1IMU estimates of angular velocity. The authors concluded that to assess wheelchair rotational angular velocity, and other metrics requiring these variables, a 3IMU solution is required. These are important comparative studies demonstrating the potential inconsistencies of using the Madgwick-based 1IMU approach [4,5]. It is important to note that the oscillating error seen by van Dijk et al. (2021) may in fact be due to sensor mounting misalignment, and the correction outlined in 2.4.3 of this study may improve wheelchair kinematic estimates. Additionally, further refinements to the Madgwick orientation filter or the use of other sensor fusion orientation filters might improve the accuracy of this approach [17].

There exist multiple sensor fusion orientation filters that may be suitable to being implemented for an accurate 1IMU wheelchair sports solution [13,17,18]. However, while these filters are developed to handle the complex 3D orientation of the sensor in universal contexts, the solution presented here is merely a simplified approach to the unique determination of wheelchair frame rotation in wheelchair court sports that are performed on a level surface. Therefore, this solution may be limited to this specific use case and may not be able to be applied in other wheelchair applications [18]. However, this solution may also benefit through constraining the criteria as well as sensors and axis used. By limiting the degrees of freedom required at separate computational phases as well as capitalizing on a complimentary two-axes wheel orientation solution, a simple, accurate and repeatable solution has been developed. Two important preliminary steps in this process, correcting for camber angle and IMU mounting misalignment, should be accounted for within any suitable orientation filter but seemed to not be handled consistently by previous Madgwick 1IMU implementations [11,12]. This demonstrates a potential limitation in using a comprehensive orientation filter where errors from other sensors and axes could be introduced or not properly accounted for. Further, the use of the two-argument arctangent function with the wheel plane accelerometer gravitational signals alone produces a very stable and useful orientation estimate that could not have occurred with a single axis alone and could be complicated by including more degrees of freedom [3]. The use of the two-argument arctangent function in this solution removes the requirement of gyroscope and magnetometer data from the orientation estimation which can result in orientation drift through the integration of the gyroscope data and potential errors due to changes in the magnetic field. Taken together, the novel, constrained and sequential approach presented here may provide a suitable estimate of wheelchair rotational kinematics and may reduce the requirement of a 3IMU solution.

Van der Slikke et al. (2015) truly revolutionized wheelchair athlete monitoring when they presented a 3IMU solution which is now considered the standard for deriving valid and reliable wheelchair mobility metrics [3]. However, the requirement of 3IMU hardware and software solutions is not universally accessible, and a 1IMU solution could provide a great opportunity that is more costly and feasible for wheelchair athletes at all levels of competition as well as daily wheelchair users. The computational approach presented here has the potential to support a 1IMU wheelchair implementation as the wheelchair frame rotation estimation is comparable to the 3IMU solution as the direct measurement of the frame-mounted IMU gyroscope. There remains a need to use this approach to derive multiple linear and rotational wheelchair kinematics across various training and competition conditions as well as athlete cohorts and confirm the suitability of this approach against the 3IMU standard. As an important next step, wheelchair velocity as calculated using the 3IMU approach can be compared to wheelchair velocity using one wheel-mounted IMU. While the algorithms required to calculate wheelchair velocity using the 1IMU approach need to be developed, the rotation estimate now fully supports this opportunity as well as other important metrics.

## Figures and Tables

**Table 1:** Goodness-of-fit statistics.

IMU Wheel	Left			Right		
Goodness of Fit	r^2^	RMSE (rad/s)	MAE (rad/s)	r^2^	RMSE (rad/s)	MAE (rad/s)
Mean STDEV	0.996 0.005	0.067 0.029	0.049 0.022	0.995 0.003	0.074 0.024	0.055 0.018

## Data Availability

Data used in this study were shared by Wheelchair Rugby Canada and are not available for further sharing.
